# Effects of MVA85A vaccine on tuberculosis challenge in animals: systematic review

**DOI:** 10.1093/ije/dyw058

**Published:** 2016-05-11

**Authors:** Helen McShane, Mark Hatherill, Willem Hanekom, Tom Evans

**Affiliations:** ^1^ The Jenner Institute, University of Oxford, Old Road Campus Research Building, Roosevelt Drive, Oxford OX3 7DQ,; ^2^ South African TB Vaccine Initiative, University of Cape Town, Wernher & Beit South Building, Room S 2.01, Health Sciences Faculty, Anzio Rd, Observatory, Cape Town, 7935,; ^3^ Bill and Melinda Gates Foundation, 500 Fifth Avenue North, Seattle, WA 98109 and; ^4^ Aeras, 1405 Research Blvd. Rockville, MD USA 20850

We write in response to the article by Kashangura *et al.* who conducted a systematic review of the animal efficacy data concerning MVA85A, a candidate TB vaccine. ^1^

The decision to proceed with an infant efficacy trial was based on safety and promising immunogenicity data in humans. The infant efficacy trial was preceded by a programme of 11 Phase 1-2a clinical trials of MVA85A, descending in age from adults, adolescents and children to infants. Ethical permissions and regulatory approvals were based on these and all the other existing data, published and unpublished, including results of preclinical animal experiments.

We agree that there is a need for better preclinical animal models with which to evaluate candidate TB vaccines. These models are not yet standardized. Further, there are no uniform, rigorous criteria for selecting which vaccine candidates should progress from animal models into clinical trials. ^2^ Global efforts to develop the best models and to establish standardization and gating criteria are currently underway as part of European Commission- and Bill & Melinda Gates Foundation-funded efforts. Even with predictive animal models, TB vaccine development will remain a largely iterative process. As data from human efficacy trials become available, there will be an opportunity to review the predictive value of the criteria used from animal results to select candidate vaccines for clinical testing.

It is important to recognize that, particularly within academia, product development is not the only goal of animal experimentation. Depending on the primary aim of these studies, the timing of and decision to proceed with clinical trials may often be only partially dependent on the results generated by animal models.

We are all committed to open and timely publication of results, including those that are not positive. A clear example of this was that we published the results of the South African efficacy trial just ten days after receiving the data ^3^^,^^4^ . Importantly, although MVA85A did not enhance protection against TB disease above what was afforded by BCG, this study showed that the vaccine was safe in infants, as predicted by preclinical toxicology and Phase 1/2a clinical studies of the candidate.
